# The specific features of the thyroid hormone receptor gene* THRB *polymorphism in indigenous populations of Siberia

**DOI:** 10.18699/vjgb-26-07

**Published:** 2026-03

**Authors:** B.A. Malyarchuk, N.V. Pokhilyuk, G.A. Denisova, A.N. Litvinov

**Affiliations:** Institute of Biological Problems of the North of the Far Eastern Branch of the Russian Academy of Sciences, Magadan, Russia; Institute of Biological Problems of the North of the Far Eastern Branch of the Russian Academy of Sciences, Magadan, Russia; Institute of Biological Problems of the North of the Far Eastern Branch of the Russian Academy of Sciences, Magadan, Russia; Institute of Biological Problems of the North of the Far Eastern Branch of the Russian Academy of Sciences, Magadan, Russia

**Keywords:** THRB gene, human populations, Siberia, cold adaptation, thyroid system, adaptive thermogenesis, ген THRB, популяции человека, Сибирь, адаптация к холоду, тиреоидная система, адаптивный термогенез

## Abstract

In the process of adaptation to cold in humans, genes belonging to the thyroid system signaling pathways that regulate thermogenesis, energy expenditure, and metabolic rearrangements are implicated. One such gene is the THRB gene, which encodes the nuclear receptor TRβ, with which the thyroid hormone triiodothyronine (T3) interacts. The activity of thermogenin UCP1 is influenced by the concentration of TRβ-T3 complexes, which serve to uncouple oxidative phosphorylation in mitochondria, thereby enhancing heat production. Consequently, thyroid hormone receptors have been demonstrated to play a significant role in adaptive thermogenesis. In the present study, we conducted a comprehensive analysis of published data on the THRB gene polymorphism in Siberian indigenous populations, with the objective of identifying potential associations between polymorphism variants and adaptation to cold. The analysis of exon and adjacent noncoding regions of the THRB gene revealed a single nucleotide substitution in the protein-coding region (synonymous substitution in the locus rs3752874). All other nucleotide substitutions were detected primarily in 3’-untranslated regions and introns. Analysis of the THRB haplotype distribution revealed two Koryak-specific haplotypes characterized by the rs762175401-A substitution. The results of population screening demonstrated that this substitution is prevalent among the Koryak population, with a frequency of 13.8 %, and is also present in the Siberian Eskimo population. However, in other global populations, the frequency of the rs762175401-A substitution does not exceed 0.05 % (in the Japanese and Koreans) or has even lower values (less than 0.02 %). The analysis of the nucleotide sequence of the THRB gene indicates that the rs762175401 locus is situated in the 3’-untranslated region at position +2 from the terminating codon. It is plausible that this substitution may have led to alterations in translation termination efficiency. In the case of enhanced termination efficiency, it is conceivable that it contributed to an elevated rate of protein synthesis, thereby resulting in an increase in the concentration of TRβ-T3 complexes. The higher frequency of the rs762175401-A variant in the Koryak and Eskimo populations, representing the oldest populations of Northeastern Siberia, is assumed to be due to long-term adaptation of these populations to cold.

## Abstract (ru)

В процессах адаптации к холоду у человека задействованы гены, входящие в сигнальные пути тиреоидной системы, которыми регулируются термогенез, энергетические затраты и метаболические перестройки. Один из таких генов – THRB, кодирующий ядерный рецептор TRβ, с которым взаимодействует гормон щитовидной железы – трийодтиронин (T3). От концентрации комплексов TRβ-T3 зависит активность термогенина UCP1, с помощью которого происходит разобщение окислительного фосфорилирования в митохондриях и усиливается производство тепла. Таким образом, рецепторы тиреоидных гормонов играют важную роль в адаптивном термогенезе. В настоящей работе нами впервые проведен анализ опубликованных данных о полиморфизме гена THRB в популяциях коренного населения Сибири с целью поиска вариантов полиморфизма, потенциально связанных с адаптацией к холоду. Анализ полиморфизма экзонов и прилегающих некодирующих участков гена THRB показал наличие всего одной нуклеотидной замены в белок-кодирующей области (синонимичная замена в локусе rs3752874), все остальные нуклеотидные замены выявлены преимущественно в 3’-нетранслируемых участках и интронах. Анализ распределения гаплотипов гена THRB позволил обнаружить два специфичных для коряков гаплотипа, характеризующиеся заменой rs762175401-A. Популяционный скрининг показал, что эта замена распространена среди коряков с частотой 13.8 %, а также присутствует у сибирских эскимосов, хотя в остальных группах населения мира частота замены rs762175401-A не превышает 0.05 % (у японцев и корейцев) или имеет еще более низкие значения (менее 0.02 %). Анализ нуклеотидной последовательности гена THRB показывает, что локус rs762175401 находится в 3’-нетранслируемой области в позиции +2 от терминирующего кодона. Вполне вероятно, что эта замена могла привести к изменениям в эффективности терминации трансляции и в случае повышения эффективности терминации способствовала увеличению скорости синтеза белка и, соответственно, концентрации комплексов TRβ-T3. Предполагается, что повышение частоты варианта rs762175401-A у коряков и эскимосов, представляющих древнейшее население Северо-Востока Сибири, обусловлено долговременной адаптацией популяций к холоду.

## Introduction

Physiological studies have shown that the indigenous peoples of Siberia have an increased metabolic rate, especially in winter, and changes in thyroid hormone levels synchronous with the type of metabolism (Leonard, 2024). In Yakuts, the indigenous inhabitants of one of the coldest regions of the world, metabolic heat production increases by an average of 6 % in winter (Leonard et al., 2014). In winter, the body’s tissues – mostly brown adipose tissue (BAT) – absorb thyroid hormones more actively. This increases heat production, leading to a significant drop in blood levels of triiodothyronine (T3) and thyroxine (T4) (Levy et al., 2013; Nikanorova et al., 2023). It is well established that thermogenin UCP1 (uncoupling protein-1) is expressed in BAT, where it facilitates uncoupling oxidative phosphorylation in mitochondria and heat release (Bianco, Silva, 1988). UCP1-mediated thermogenesis is activated by the interaction of thyroid hormones with the nuclear receptor TRβ. Higher concentrations of TRβ-T3 complexes result in higher UCP1 activity (Martinez de Mena et al., 2010; Lee et al., 2012; Yau, Yen, 2020; Ma et al., 2023). Thyroid hormone receptors clearly play an important role in the nonshivering thermogenesis associated with adaptation to cold.

It is well established that the distribution of polymorphism variants of the UCP1, UCP2, and UCP3 uncoupling protein genes in human populations is associated with a number of natural and climatic factors, including geographic latitude, altitude, and the severity of natural conditions (Hancock et al., 2011; Nikanorova et al., 2021, 2022; Kozlov et al., 2024).

The polymorphism of the THRB gene encoding the nuclear receptor TRβ has been characterized in various genetic databases (dbSNP, https://www.ncbi.nlm.nih.gov/snp/). However, we did not find any special publications devoted to the analysis of the distribution of polymorphic variants of this gene in human populations. The main publications related to the polymorphism of the THRB gene focus on the search for genetic variants associated with thyroid hormone resistance syndrome (Dumitrescu, Refetoff, 2013), the risk of cancer (González- Sancho et al., 2003), and the regulation of transcription and chromatin remodeling (Grøntved et al., 2015).

The present work aims to characterize the polymorphism of the THRB gene in the indigenous populations of Siberia and to search for polymorphism variants associated with adaptation to cold.

## Materials and methods

Data on whole exome-wide polymorphisms in indigenous populations of Northeastern Siberia (Eskimos, Chukchi, Koryaks; N = 25), Central Siberia (Evens, Evenks, Yakuts; N = 29), Southern Siberia (Tuvinians, Shorians, Altaians, Buryats; N = 28), and Western Siberia (Kets, Khanty, Mansi, Selkups, Nenets, Nganasans; N = 20), with the total number of 102 individuals, were used (Pagani et al., 2016). We analyzed the polymorphism of all exons and adjacent noncoding regions of the THRB gene located on chromosome 3 between positions 24158651 and 24536773. We used the ELB algorithm (Excoffier et al., 2003) implemented in the Arlequin 3.5 software package to identify haplotypes from genotypes with unknown gametic phase. Fisher’s exact test was applied to assess the statistical significance of differences in the frequencies of polymorphic variants. The median network of THRB gene haplotypes was constructed using the Network 10.2 program (www.fluxus-engineering.com). This work used information from genomic databases in human populations for comparative analysis: dbSNP (www.ncbi.nlm.nih.gov/projects/SNP), 1000 Genomes (https://www.internationalgenome.org/), and gnomAD (https://gnomad.broadinstitute.org/).

We performed population screening of polymorphisms in loci rs762175401 (nucleotide position 3:24164373) and rs72619908 (position 3:24164268) of the THRB gene. Nucleotide numbers are given according to the human genome reference sequence GRCh37.p13 (hg19). The study used total DNA isolated from whole blood from representatives of the indigenous populations of the Severo-Evensky District of the Magadan Region. The samples include 98 Koryaks and 110 Evens. The questionnaire data show that the surveyed Koryaks and Evens have identified themselves as members of the above ethnic groups for at least two to three generations.

The nucleotide sequence of the THRB gene including loci rs762175401 and rs72619908 was amplified using oligonucleotide primers 5ʹ-GCGCCATTTTGCTGACTCAA-3ʹ and 5ʹ-TCTTCTCTCTTCCCCGCAGA-3ʹ. Primers were designed based on the nucleotide sequence of the THRB gene (number NC_000003.12 in the GenBank database) using the Primer3 program (Untergasser et al., 2012).

Amplification products were sequenced using the Brilliant- Dye™ Terminator Cycle DNA Sequencing kit v3.1 (Netherlands) and an ABI Prism 3500xL genetic analyzer (Applied Biosystems, USA). MEGA5 package programs were used for nucleotide sequence alignment and analysis (Tamura et al., 2011). We calculated the allele frequencies, heterozygosity, and correspondence of the genotype distribution to Hardy– Weinberg equilibrium using the Arlequin 3.5 software package (Excoffier, Lischer, 2010).

## Results and discussion

The analysis of the THRB gene nucleotide sequences from 102 individuals belonging to different ethnic groups within the indigenous Siberian population revealed polymorphisms at 22 nucleotide positions (Table 1). However, only one polymorphism variant was detected in the exons: a synonymous substitution at locus rs3752874 (amino acid position 245). All other polymorphisms were found in the non-coding region, primarily in the 3ʹ-untranslated regions and introns of the gene. Analysis of the distribution of polymorphic variants revealed that a similar distribution of allele frequencies was observed only in two cases (rs56204436-A and rs13326381-T) in Siberia, East Asia, and Europe (p > 0.05)

**Table 1.**
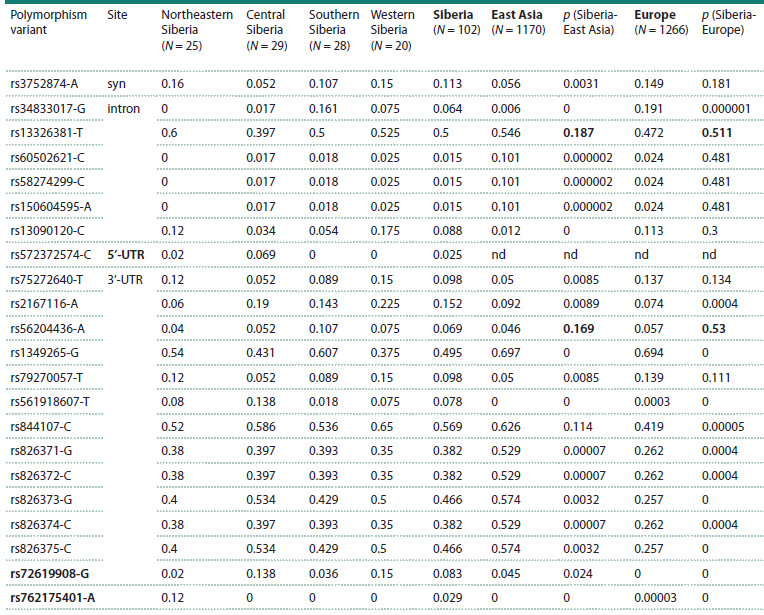
Frequencies of THRB gene polymorphism variants in indigenous populations of Siberia, East Asia and Europe Note. Data for East Asian and European populations are from the 1000 Genomes (https://www.internationalgenome.org/) and gnomAD (https:// gnomad.broadinstitute.org/) databases. The statistical significance of differences (p) between the frequencies of polymorphism variants in the compared regions was evaluated using Fisher’s exact test. Designations: 3’-UTR and 5’-UTR – 3’- and 5’-untranslated regions; syn – synonymous substitution in the protein-coding region; nd – no data. The polymorphism variants for which additional population screening was performed in the present study, as well as p-values greater than 0.05 in cases with a similar allele frequency distribution in Siberia, East Asia, and Europe (for rs56204436-A and rs13326381-T), are highlighted in bold.

In most cases, there are statistically significant differences in the frequency of polymorphic variants in Siberia compared to Europe and East Asia. For several loci, the allele frequencies of Siberian populations are more similar to European than East Asian values (rs75272640, rs79270057, rs3752874, rs60502621, rs58274299, rs150604595, rs13090120). In three cases, polymorphisms characteristic of Siberian populations were detected in all four regions (rs561918607-T) or in particular populations (rs572372574-C in Chukchi and Evenki, and rs762175401-A in Eskimos and Koryaks) (Table 1).

To analyze the distribution of THRB gene haplotypes in Siberian populations, we narrowed down the set of polymorphic loci to 16: rs75272640, rs2167116, rs56204436, rs1349265, rs79270057, rs561918607, rs844107, rs826371, rs72619908, rs762175401, rs3752874, rs572372574, rs34833017, rs13326381, rs60502621, rs13090120. This was done because several loci located close to each other showed similar frequencies in Siberian, East Asian, and European samples and were linked. The ELB algorithm identified 34 16-locus haplotypes (Table S1 of the Supplementary Material)1. For the subsequent phylogenetic analysis of the haplotypes, we used the 22 haplotypes that were recorded more than once in the populations.

Supplementary Materials are available in the online version of the paper: https://vavilov.elpub.ru/jour/manager/files/Suppl_Mal_Engl_30_1.xlsx


The median network of haplotypes obtained demonstrates rather complex phylogenetic relationships, likely due to recurrent mutations in the non-coding regions of the THRB gene or sequencing errors at the rs13326381 locus (see the Figure). The haplotypes under study were detected in different regional groups in Siberia; therefore, geographic clustering of haplotype groups is not apparent. Of particular interest are the HT19 and HT20 haplotypes found in samples from Northeastern Siberia. These haplotypes are characterized by the presence of a G→A substitution at locus rs762175401, which is located in the 3ʹ-untranslated region of the gene. This variant polymorphism was only found in Eskimos (25 %) and Koryaks (12 %). According to the dbSNP and gnomAD data, the rs762175401- A allele was detected at very low frequencies in the Japanese (0.042 %) and Koreans (0.04 %), and in large samples from East Asia (0.013 %), the Middle East (0.016 %), South Asia (0.005 %), and Europe (0.002 %).

**Fig. 1.**
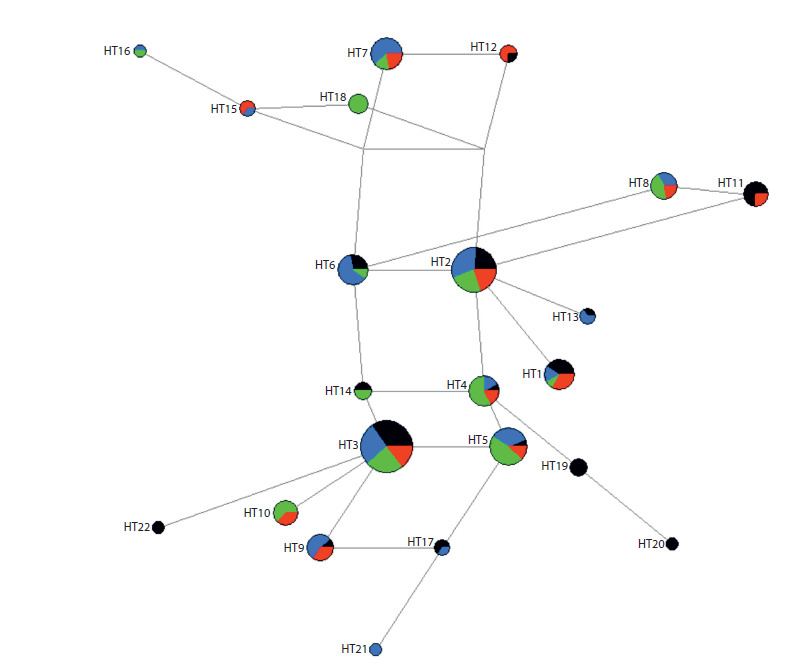
Median network of THRB gene haplotypes in the indigenous Siberian populations Black indicates Northeastern Siberia, blue – Central Siberia, green – Southern Siberia, red – Western Siberia.

Since the Siberian sample size was insufficient, we investigated the polymorphism of the rs762175401 locus in a more representative sample of Koryaks and Evens from the Magadan region. We also included the rs72619908 locus, located 104 bp from rs762175401, in the investigated region of the THRB gene (Tables 2 and 3). According to historical data, the Koryaks (along with the Chukchi) belong to north-eastern Paleoasians, the oldest population in Northeastern Siberia. The Tungus-speaking Even people began settling in Koryak areas around the 17th century (Khakhovskaya, 2024). However, despite living in the same neighborhood for a long time and intermarrying, these ethnic groups have retained features of their gene pools that are adapted to varying degrees to the extreme conditions of their natural environment (Cardona et al., 2014; Derenko et al., 2023; Malyarchuk, Derenko, 2024).

**Table 2.**
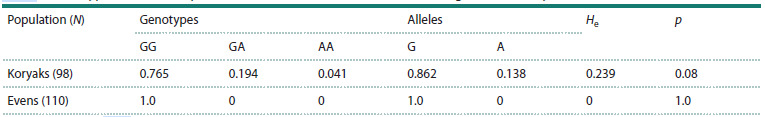
Genotype and allele frequencies of the rs762175401 locus of the THRB gene in the Koryaks and Evens Note. Here and in Table 3: N – sample size, He – expected heterozygosity, p – statistical significance of deviation from Hardy–Weinberg equilibrium (significant at p <0.05).

**Table 3.**
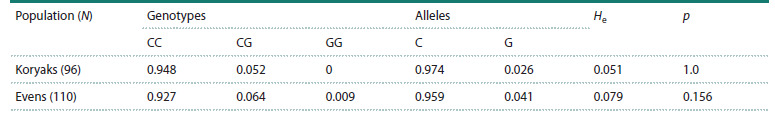
Genotype and allele frequencies of the rs72619908 locus of the THRB gene in the Koryaks and Evens

Analysis of the rs762175401 polymorphism showed that, of the studied samples, only Koryaks have the rs762175401-A allele, which occurs at a frequency of 13.8 % (Table 2). The polymorphic variants of the rs72619908 locus are distributed similarly in both ethnic groups (Table 3). The high frequency of the rs762175401-A variant in Koryaks, as well as its presence in Siberian Eskimos, suggests that this polymorphism has adaptive significance with respect to adaptive thermogenesis. The rs762175401 locus is located in the 3ʹ-untranslated region of the THRB gene, at position +2 relative to the UAG termination codon. A substitution at this nucleotide position could potentially alter translation termination efficiency, as the 3ʹ context of stop codons has been shown to influence translation termination in eukaryotes (Cridge et al., 2018; Sokolova et al., 2020). If the rs762175401-A substitution contributes to increased termination efficiency, it could lead to a higher rate of protein synthesis, thus optimizing translation (increasing mRNA stability, ribosome recycling, and translation fidelity), as was previously found for Escherichia coli and yeast (Baggett et al., 2017; Wu et al., 2020).

Improved thyroid hormone receptor synthesis presumably contributes to an increased concentration of TRβ-T3 complexes and may therefore have adaptive significance in cold conditions. Thus, the increased frequency of the rs762175401-A variant in the Koryak and Eskimo populations, which are among the oldest in Northeastern Siberia, can be explained by the long-term influence of climatic factors on THRB gene function.It is known that several genes associated with cold adaptation in humans, including DIO2, UCP1, UCP3, THRB, PPARGC1A, and RXRA, are involved in thyroid signaling pathways that regulate thermogenesis as well as energy expenditure and metabolic rearrangements (Laurberg et al., 2005; Bianco et al., 2019; Tsibulnikov et al., 2020). Genetic studies have revealed population genetic effects with respect to certain genes, such as UCP1 and UCP3, which are manifested by an increased frequency of specific polymorphism variants in Northeast Asia (Nikanorova et al., 2021, 2022; Kozlov et al., 2024). Chronic exposure to cold in Arctic populations likely increases the activity of the DIO2 enzyme, which regulates T3 levels in cells. This increases the production of T3 to compensate for the high metabolic demands of thermogenesis (Noahsen et al., 2021).

Natural selection has likely affected the ANGPTL8 and PLA2G2A genes in indigenous Siberian peoples, such as the Koryaks, Yukaghirs, and Nenets (Hallmark et al., 2019). These genes are involved in the adaptive response to cold; they are activated by hormone T3 and participate in regulating lipid metabolism (Sharma et al., 2014; Tseng et al., 2014). The effect of selection on the THRB gene has also been reported in the Yakut sample using the PBS test (Cardona et al., 2014). However, an analysis of the effect of selection in regional groups worldwide found statistical associations with thyroid system function, BAT, and thermoregulation only in Central Siberians and Africans using the EHH test (Pagani et al., 2016).

However, a recent genomic study of Greenland Eskimos did not reveal selection acting on thyroid system genes (Stæger et al., 2025). Polymorphism variants specific to Greenlandic and North American Eskimos as well as Siberian populations (FADS1/2, SI, CPT1A, TBC1D), or only to Greenlandic and American Eskimos (LDLR, HNF1A, ADCY3, ATP8B1, PCCA/ PCCB) were found mainly in lipid and carbohydrate metabolism genes (Stæger et al., 2025).

These data suggest the necessity of further investigating thyroid system gene polymorphisms in indigenous populations of the Far North of various ethnical backgrounds, as adaptive changes in gene pools may exhibit population-specific characteristics.

## Conclusion

Thus, our study provided the first estimate of the prevalence of different THRB gene polymorphisms that encode the thyroid hormone receptor in ethnic groups of the indigenous Siberian population. Our analysis of THRB gene haplotype distribution revealed that the rs762175401-A variant, observed in Koryaks (at a frequency of 13.8 %) and Siberian Eskimos (25 %), is prevalent in Northeastern Siberia. The increase in the frequency of this polymorphism is likely associated with the adaptation of indigenous Far North populations to cold and with rearrangements of the thyroid system, which is directly involved in nonshivering thermogenesis. Further molecular genetic studies will help clarify these mechanisms

## Conflict of interest

The authors declare no conflict of interest.
